# Occupational characteristics and high-risk pregnancy outcomes among Korean nurses: a secondary analysis of the Korea Nurses’ Health Study

**DOI:** 10.4069/whn.2026.08.19

**Published:** 2026-09-29

**Authors:** Juyoung Ha, Minji Kim

**Affiliations:** College of Nursing, Pusan National University, Yangsan, Korea

**Keywords:** Nurses, Pregnancy, Secondary data analysis, Shift work schedule, Working conditions

## Abstract

**Purpose:**

This study examined associations between multidimensional occupational characteristics and high-risk pregnancy outcomes among nurses.

**Methods:**

This cross-sectional secondary analysis used Korea Nurses’ Health Study data collected from 2015 to 2019. Occupational characteristics included shift work type, hospital bed size, overtime work, prolonged standing, heavy lifting, and emotional labor. Nine self-reported outcomes were assessed: preterm birth, preterm labor, preeclampsia/eclampsia, gestational diabetes mellitus, incompetent internal os of the cervix, placenta previa, placental abruption, oligohydramnios/polyhydramnios, and premature rupture of membranes. Multivariable logistic models simultaneously included demographic, occupational, and reproductive health factors; Firth penalization was used for sparse outcomes.

**Results:**

Among 1,330 nurses, 748 (56.2%) reported at least one high-risk pregnancy outcome. Frequencies did not differ significantly by shift work type. In adjusted models, emotional labor was associated with preterm labor; hospital bed size with gestational diabetes mellitus; heavy lifting with preterm birth and placental abruption; and standing time with preterm birth. Endometriosis history was associated with preterm labor, and uterine myoma history with preterm labor, preterm birth, and placenta previa.

**Conclusion:**

Selected occupational and reproductive health factors, rather than shift work type alone, were associated with high-risk pregnancy outcomes in the fitted models. Because occupational exposure timing may not have corresponded to the most recent pregnancy and the outcomes were self-reported, the findings require cautious interpretation and prospective confirmation before they guide pregnancy-specific risk assessment or work modification.

## Introduction

High-risk pregnancy is an important issue in women’s and occupational health because it can adversely affect maternal health, psychological well-being, and postpartum outcomes. In South Korea, the mean maternal age at childbirth was 33.69 years in 2024. The proportion of primiparous women aged 35 years or older increased from 23.9% in 2015 to 35.9% in 2024 [1]. Pregnancy at age 35 years or older is generally classified as advanced maternal age and is a major risk factor for high-risk pregnancy [2]. Multiple pregnancy associated with assisted reproductive technology and a history of gynecologic disease or surgery may also increase risk [2]. High-risk pregnancy reflects interactions among biological, psychological, social, and environmental factors [3]. Biological factors include advanced maternal age, multiple pregnancy, and gynecologic history [2], whereas psychological and social factors include stress-related vulnerabilities affecting maternal well-being [3,4]. Occupational and environmental conditions may further contribute to pregnancy-related health risks. Identifying factors associated with high-risk pregnancy outcomes and occupational groups potentially vulnerable to these outcomes is therefore important for women’s and occupational health.

Nurses constitute a particularly relevant occupational group for investigating high-risk pregnancy outcomes. Nursing is a female-dominated profession, and many nurses continue to provide direct patient care during their reproductive years. Among Korean nurses, previous studies have documented concerns related to shift-schedule patterns [5], dietary behaviors [6], fatigue and sleep-related problems [7], and premenstrual symptoms [8]. These findings indicate that shift work occurs within a broader context of occupational and health-related demands. Clinical nursing involves multiple concurrent demands, including irregular schedules, overtime, prolonged standing, heavy lifting, emotional labor, and occupational stress [9,10]. Nurses may also be exposed to infectious agents, disinfectants, antineoplastic agents, and radiation, many of which arise during direct patient care and may involve repeated exposure within a single shift [10,11]. Thus, nursing provides a distinct occupational context in which physical, psychosocial, organizational, and hazardous exposures may coexist and potentially influence reproductive and pregnancy-related health.

Research in nursing populations further supports the need to examine these occupational risks comprehensively. Hino et al. [9] found that pregnant nurses had higher occupational stress and job demands, as well as lower job control, than pregnant workers in other occupations. Nurse-specific factors also included fewer opportunities to take breaks and difficulty prioritizing fetal health [9]. In a nationwide study of Chinese nurses, Jiang et al. [10] evaluated multiple occupational exposures, including night work, long working hours, prolonged standing, heavy lifting, and exposure to antineoplastic agents. Selected occupational exposures were associated with adverse pregnancy outcomes [10]. In addition, a meta-analysis found that occupational exposure to antineoplastic agents among nurses was associated with increased risks of spontaneous abortion, stillbirth, and congenital abnormalities [11]. Taken together, these findings indicate that nurses’ reproductive health cannot be adequately understood by examining shift work alone.

Evidence from broader working populations also suggests that occupational characteristics may affect pregnancy outcomes. Evening shift work has been associated with gestational diabetes mellitus (GDM) [12]. Night shift work among midwives has been linked to infertility and miscarriage [13]. Long working hours and high physical workload during pregnancy have also been associated with adverse outcomes, including preterm birth [14]. However, these studies generally examined specific work schedules, occupational groups, or pregnancy outcomes. Their findings may therefore not fully represent the multiple, overlapping exposures encountered in clinical nursing.

Previous research involving nurses has also tended to focus on a single occupational exposure or a limited set of reproductive outcomes [9-11]. As a result, the independent associations of work schedules, physical workload, emotional labor, and organizational conditions with high-risk pregnancy outcomes remain unclear. Few studies have examined these occupational characteristics simultaneously while also accounting for demographic and reproductive health factors. A comprehensive analysis is therefore needed to identify occupational risks that may be amenable to pregnancy-related risk assessment and individualized work adjustment. Such evidence may help nurse managers and health care organizations develop workplace protections suited to pregnant nurses.

Accordingly, this study examined associations between multidimensional occupational characteristics and high-risk pregnancy outcomes among Korean female nurses.

## Methods

**Ethics statement:** This secondary analysis used de-identified data from the Korea Nurses’ Health Study. The original data were collected with prior informed consent from participants under Institutional Review Board approval. The present study was approved by the Institutional Review Board of Pusan National University with a waiver of informed consent (IRB/2025_73_HR). Clinical & Omics Data Archive (CODA) also approved data access, and all analyses were conducted in accordance with CODA data-use and security requirements.

### Study design

This study used a cross-sectional correlational design and secondary data from the Korea Nurses’ Health Study (KNHS). The KNHS is an ongoing prospective cohort study initiated in 2013 to examine associations among occupational, environmental, lifestyle, and health-related factors in Korean female nurses. For this purpose, the present secondary analysis used fourth-wave raw data collected from 2015 to 2019 and provided through CODA. Detailed information on the overall KNHS design, participant recruitment, and data-collection procedures has been described previously [15]. This report followed the Strengthening the Reporting of Observational Studies in Epidemiology guideline for cross-sectional studies [16].

### Participants

The KNHS began as a prospective cohort of Korean nurses aged 20–45 years who self-reported female sex. Because the present study examined pregnancy-related outcomes, the analytic sample was restricted to participants reporting a current pregnancy or a pregnancy since 2013, with further exclusions described below.

Of 10,636 nurses eligible for the fourth-wave survey, 9,267 participated in the survey. The study excluded 7,559 who reported neither a pregnancy since 2013 nor a current pregnancy, leaving 1,708 participants for descriptive analyses. This group comprised 327 participants who were currently pregnant and 1,381 who reported a pregnancy since 2013. A further 378 participants with missing subsequent pregnancy-related or high-risk-outcome responses were excluded from regression analyses, leaving 1,330. Figure 1 presents the selection process.

A post hoc power calculation used G*Power version 3.1.9.7 (Heinrich Heine University Düsseldorf, Düsseldorf, Germany) and an odds ratio of 2.05 reported by Kader et al. [17] for working hours and preterm birth among pregnant health care employees. With 1,330 participants, a two-tailed significance level of .05, and an event probability of .047 under the null hypothesis, the reported achieved power was 89.5%, supporting the adequacy of the final sample for the primary logistic regression analysis.

### Measures

#### High-risk pregnancy outcomes

The dependent variables were nine high-risk pregnancy outcomes reported for the most recent pregnancy: preterm birth, preterm labor, preeclampsia/eclampsia (PE/E), GDM, incompetent internal os of the cervix (IIOC), placenta previa, placental abruption, oligohydramnios/polyhydramnios, and premature rupture of membranes (PROM). The report also summarized whether a participant had at least one of these outcomes.

Pregnancy-related outcomes were assessed among participants who reported that their most recent pregnancy resulted in a singleton, twin, or higher-order multiple pregnancy. Participants were asked whether they had experienced each condition during that pregnancy, using single items such as “Did you have preterm birth in your most recent pregnancy?” Responses were recorded as yes or no, and each outcome was analyzed separately as a binary variable.

#### General and reproductive health characteristics

General and reproductive health characteristics included age and histories of endometriosis and uterine myoma. Age was categorized as <35 years or ≥35 years according to the criterion for advanced maternal age [2]. Histories of endometriosis and uterine myoma were classified separately as yes or no. Consistent with the previous KNHS study [18], these histories were analyzed as dichotomous variables.

#### Occupational characteristics

Occupational characteristics included shift work status, night shift work, hospital bed size, overtime work, prolonged standing, heavy lifting, and emotional labor. The KNHS questionnaires were based on the US Nurses’ Health Study 3 questionnaires and were translated and modified by a multidisciplinary advisory group to reflect Korean working conditions and research needs [15]. In the present study, the original KNHS response categories were retained when they allowed more detailed classification of occupational exposure levels. Measurement items and categorization of the other occupational variables were informed by a previous KNHS study of occupational factors among female nurses [18]. The categorization used in the present study is described below in relation to that previous study.

Shift work status was assessed using the question, “Have you worked shifts during the past year?” Participants who responded “yes” were classified as shift workers, and those who responded “no” were classified as non-shift workers. Shift workers were then asked, “Have you worked night shifts during the past year?” Those who responded “yes” were classified as shift workers with night shifts. Accordingly, shift work type was categorized into three groups: non-shift work, shift work without night shifts, and shift work with night shifts. For additional analyses, shift work status was also dichotomized as non-shift work or shift work. The previous KNHS study primarily classified participants according to whether they performed shift work [18], whereas the present study further distinguished shift workers according to whether their schedules included night shifts, based on the original KNHS items [15].

Hospital bed size was categorized as <100 beds, 100–599 beds, or ≥600 beds. These categories were provided in the KNHS questionnaire and were retained in the present study [15].

Overtime work was assessed using average weekly overtime hours and recategorized as no overtime work or any overtime work. Participants reporting 0 overtime hours were classified as having no overtime work, whereas those reporting ≥1 hour were classified as having overtime work. The previous KNHS study categorized total weekly working hours as ≤40 or ≥41 hours [18]. In contrast, the present study focused on the presence of overtime work and categorized participants according to whether they reported any weekly overtime hours.

Prolonged standing was assessed as the number of hours spent standing during a workday and categorized as 0 to <1 hour/day, 1–4 hours/day, 4–9 hours/day, or ≥9 hours/day. The previous KNHS study categorized standing time as <1 hour/day, 1–4 hours/day, or ≥5 hours/day [18]. In the present study, the original KNHS response categories were retained; therefore, the ≥5-hour category used in the previous study was divided into 4–9 hours/day and ≥9 hours/day [15].

Heavy lifting was assessed according to the frequency of moving objects weighing ≥10 kg during a workday and categorized as none, 1–5 times/day, 6–15 times/day, or ≥16 times/day. The previous KNHS study categorized heavy lifting as none, 1–5 times/day, or ≥6 times/day [18]. In the present study, the original KNHS questionnaire response categories were retained; therefore, the ≥6-times/day category used in the previous study was further divided into 6–15 times/day and ≥16 times/day [15].

Emotional labor was measured using a single item from the original KNHS questionnaire [15]. Participants rated the severity of emotional labor in their current work from 0 (“none at all”) to 10 (“very severe”), with higher scores indicating greater emotional labor. The original KNHS score range of 0 to 10 was used as a continuous variable without categorization in the present study [15].

### Data collection

This study used preexisting cohort data and did not involve new data collection. The researchers submitted a proposal to CODA to use human-derived data and obtained approval from the data distribution committee. The fourth-wave raw KNHS data were then provided through CODA. The KNHS collected information on nurses’ health status, work environment, and reproductive health through an online survey. For the present study, variables related to occupational characteristics, pregnancy, and childbirth were extracted and analyzed.

Access to the raw data was restricted to approved researchers, and all analyses were conducted in the secure CODA remote analysis system. The analyzed data and research outputs were stored securely according to CODA requirements.

### Data analysis

Data were analyzed using SAS software (SAS Institute, Cary, NC, USA) provided through the CODA remote analysis system. Participants’ general, reproductive health, and occupational characteristics and high-risk pregnancy outcomes were summarized as frequencies and percentages for categorical variables and as means and standard deviations for continuous variables.

Differences in participant characteristics according to shift work type were analyzed using one-way analysis of variance with the Tukey post hoc test for continuous variables and the chi-square test for categorical variables. The distribution of high-risk pregnancy outcomes according to shift work type was also examined using the chi-square test.

A separate multivariable logistic model was fitted for each high-risk pregnancy outcome. Shift work type, age group, hospital bed size, overtime work, daily standing time, frequency of moving objects weighing at least 10 kg, emotional labor score, and histories of endometriosis and uterine myoma were entered simultaneously. Adjusted odds ratios (aORs) and 95% confidence intervals (CIs) were used to summarize associations.

Variables were selected a priori on the basis of the study aim and previous evidence. Age was included because maternal age of 35 years or older is a major risk factor for high-risk pregnancy [2]. Occupational characteristics were included on the basis of previous studies of occupational exposures and pregnancy outcomes [10,14] and a previous KNHS study of work-related characteristics among female nurses [18]. Histories of endometriosis and uterine myoma were included because these gynecologic conditions have been associated with adverse pregnancy outcomes [19].

Firth penalized logistic regression was applied to outcomes with fewer than 35 events to reduce small-sample bias and improve estimation.

## Results

### Participant characteristics by shift work type

A total of 1,708 nurses were included in the descriptive analysis: 883 non-shift workers, 133 shift workers without night shifts, and 692 shift workers with night shifts. Age differed significantly by shift work type (*p*<.001), and the Tukey post hoc test showed that non-shift workers were significantly older than shift workers with night shifts. The distribution of age groups (<35 and ≥35 years) also differed significantly by shift work type (*p*<.001). Hospital bed size differed significantly across shift work types (*p*<.001). Weekly overtime hours also differed significantly among the three groups (*p*<.001), with both shift work groups reporting significantly longer overtime hours than non-shift workers. Standing work hours and the frequency of moving objects weighing ≥10 kg differed significantly by shift work type (*p*<.001 for both). Emotional labor scores differed significantly among the three groups (*p*<.001), and the Tukey post hoc test showed that both shift work groups had significantly higher emotional labor scores than non-shift workers. Histories of endometriosis and uterine myoma did not differ significantly among the three groups (Table 1).

### High-risk pregnancy outcomes by shift work type

Among the 1,330 nurses included in the multivariable analyses, 748 (56.2%) reported at least one of the nine high-risk pregnancy outcomes. The frequency of no individual outcome differed significantly by shift work type. PROM was reported by 73 (9.9%) non-shift workers, 6 (5.2%) shift workers without night shifts, and 60 (12.5%) shift workers with night shifts; the overall comparison approached but did not reach statistical significance (*p*=.053) (Table 2).

### Adjusted associations of occupational and reproductive health factors with high-risk pregnancy outcomes

Table 3 presents adjusted associations of occupational and reproductive health factors with the nine outcomes. Firth’s penalized logistic regression was used for PE/E, IIOC, placenta previa, and placental abruption because each had fewer than 35 events. Shift work type, age, hospital bed size, overtime work, standing time, heavy lifting frequency, emotional labor, endometriosis history, and uterine myoma history were included simultaneously in each model.

Neither shift work type nor age was significantly associated with any outcome. Compared with nurses working in hospitals with at least 600 beds, those working in hospitals with 100–599 beds had higher odds of GDM (aOR, 1.75; 95% CI, 1.24–2.13). Overtime work was not significantly associated with any outcome. Compared with nurses who stood for at least 9 hours per day, those who stood for less than 1 hour per day had higher odds of preterm birth (aOR, 4.94; 95% CI, 3.14–5.23).

Compared with nurses who moved objects weighing at least 10 kg at least 16 times per day, those who did not perform such lifting had lower odds of preterm birth (aOR, 0.30; 95% CI, 0.11–0.86) and placental abruption (aOR, 0.09; 95% CI, 0.07–0.53). Each one-point increase in emotional labor was associated with higher odds of preterm labor (aOR, 1.11; 95% CI, 1.06–1.84). Participants without endometriosis had lower odds of preterm labor than those with endometriosis (aOR, 0.41; 95% CI, 0.33–0.64). Participants without uterine myoma had lower odds of preterm birth (aOR, 0.51; 95% CI, 0.14–0.72), preterm labor (aOR, 0.40; 95% CI, 0.12–0.93), and placenta previa (aOR, 0.36; 95% CI, 0.21–0.87) than those with uterine myoma. The Nagelkerke R² was .33.

## Discussion

Selected occupational and reproductive health factors were associated with high-risk pregnancy outcomes in this sample, whereas shift work type was not significantly associated with any of the nine outcomes. Hospital bed size, standing time, heavy lifting, emotional labor, endometriosis history, and uterine myoma history were associated with selected outcomes. These findings indicate that specific occupational demands and reproductive health factors should be assessed together when evaluating pregnancy-related risks among nurses.

Nurses who worked shifts that included night shifts were younger and reported longer overtime hours and higher emotional labor scores than non-shift workers. They were also more likely to work in hospitals with ≥600 beds. Previous studies have reported long working hours, high work intensity, exhaustion, and sleep disturbance among shift nurses [20,21]. Thus, shift work may occur alongside other occupational burdens that are not adequately captured by shift status alone.

In this study, 56.2% of nurses reported at least one high-risk pregnancy outcome. This proportion exceeded the 18.8% reported in a rural community study [22] and the 29.0% reported in a hospital-based survey [23]. A Korean national study reported preeclampsia in 1.6% of women and medication-treated GDM in 0.7% [24]. The relatively high proportion observed in the present study suggests that pregnancy-related health problems among nurses warrant attention as an occupational health issue. Pregnant workers may experience multiple physical, psychosocial, and ergonomic demands simultaneously [25], supporting the need to assess combinations of occupational exposures rather than shift work alone. Early assessment of pregnancy history and occupational exposures may therefore help identify nurses who require clinical consultation or work adjustment.

The nonsignificant association between shift work and high-risk pregnancy outcomes differs from previous findings based on more detailed exposure measurements. In a Swedish cohort study, nurses who worked more than 25 night shifts and three or more consecutive night shifts during the first trimester had a higher likelihood of preterm birth [17]. A systematic review also found associations of shift work and long working hours with preterm birth [14]. In addition, shift work and short sleep duration have been associated with adverse pregnancy outcomes [26], and night work during pregnancy has been examined in relation to small-for-gestational-age birth [27]. These studies assessed the timing, frequency, or sequence of night work more specifically than the present study did.

In contrast, the KNHS assessed whether nurses had performed shift work or night work during the previous year. This measure did not capture trimester-specific exposure, night-shift frequency, consecutive night shifts, recovery intervals, or schedule changes during pregnancy. Korean labor regulations also restrict overtime, night, and holiday work during pregnancy and permit transfer to lighter duties [28]. Pregnant nurses may therefore have reduced night work or changed duties, which could have attenuated differences between exposure groups. Future studies should use detailed work-schedule data to assess the timing, frequency, and sequence of night shifts together with pregnancy-related changes in work assignments.

Advanced maternal age is associated with increased risks of adverse pregnancy outcomes [2,29]. In this study, however, age was not significantly associated with any individual high-risk pregnancy outcome. The relatively restricted age distribution and the small number of nurses aged ≥35 years in some shift-work groups may have limited the ability to detect age-related differences. Future studies should include broader age distributions and sufficient numbers of nurses aged ≥35 years to clarify age-related differences in pregnancy outcomes.

Hospital bed size was associated with GDM, as nurses who worked in hospitals with 100–599 beds had higher odds of GDM than those who worked in hospitals with ≥600 beds. This finding may reflect differences in health care environments or clinical processes associated with hospital size rather than an effect of bed size itself. GDM case identification can vary according to the screening and diagnostic approach used [30,31], and current clinical guidance recognizes more than one diagnostic strategy [31]. Therefore, differences in screening or diagnostic practices across health care settings may have contributed to the observed association. Referral patterns for high-risk pregnancies may also differ across health care settings, although this possibility was not within the scope of the present study. Further studies incorporating clinical and hospital-level information are needed to clarify the association between hospital size and GDM.

Standing for less than 1 hour per day was associated with higher odds of preterm birth than standing for at least 9 hours per day. This direction contrasts with evidence linking prolonged standing and greater physical workload to adverse pregnancy outcomes [14,32]. Standing time alone may not represent total physical workload, and pregnancy-related reassignment or reduction of duties could produce reverse causation. Moreover, the recorded work schedule may not have coincided with the pregnancy. Prospective studies should measure standing, other physical demands, rest periods, and changes in duties during each trimester.

Heavy lifting was associated with preterm birth and placental abruption. Participants who did not move objects weighing at least 10 kg had lower odds of these outcomes than those who reported such lifting at least 16 times per day. This pattern is consistent with evidence linking physical workload during pregnancy to adverse outcomes [14,32]. Patient transfer and repositioning are common sources of physical load in nursing [33,34]. These findings suggest that task-specific physical demands, such as repeated heavy lifting, may be particularly important when assessing occupational risks during pregnancy. Hospitals should therefore assess lifting and patient-handling frequency and consider temporary modification of repeated lifting tasks for pregnant nurses.

Higher emotional labor scores were associated with preterm labor. Nursing requires emotional regulation during interactions with patients, families, colleagues, and other health professionals [35], and emotional labor has been associated with stress, burnout, and lower job satisfaction [35]. The present result identifies a psychosocial correlate for prospective study; it does not establish a mechanism or a pregnancy-specific causal effect because emotional labor was rated for current work.

Reproductive health history was also relevant. Nurses without a history of endometriosis had lower odds of preterm labor, whereas a history of uterine myoma was associated with preterm birth, preterm labor, and placenta previa. These findings are consistent with previous evidence linking endometriosis and uterine myoma with adverse pregnancy outcomes [19,36]. They also suggest that occupational risk assessment during pregnancy should incorporate reproductive health history as well as current occupational exposures.

This study has several limitations. First, because this was a secondary analysis of KNHS data, detailed information on trimester-specific shift work, night-shift frequency, sleep, unit workload, and pregnancy-related work adjustment was unavailable. Second, complete-case analysis may have introduced selection bias, and small event numbers for some outcomes limited statistical power and estimate stability, although Firth’s penalized logistic regression was applied. Third, pregnancy outcomes were self-reported rather than verified using medical records; therefore, recall bias and misclassification cannot be excluded. Fourth, the cross-sectional observational design precludes causal inference. Fifth, data collected from 2015 to 2019 may not fully reflect current nursing environments because staffing, work schedules, labor policies, and pregnancy-support practices may have changed since the study period.

Despite these limitations, this study suggests that high-risk pregnancy outcomes among nurses may reflect multiple occupational and reproductive health factors rather than shift work alone. The findings highlight the importance of considering specific physical demands, psychosocial workload, and reproductive health history together, while recognizing that the present exposure timing does not establish pregnancy-specific risk. Future prospective studies should align detailed trimester-specific exposures with clinically verified outcomes, account for pregnancy-related work changes and major confounders, and prespecify outcome-specific models and multiplicity procedures. Such studies should also examine exposure timing, dose-response relationships, and changes in work assignments during pregnancy.

In conclusion, shift work type was not significantly associated with high-risk pregnancy outcomes among Korean female nurses. However, hospital bed size, standing work hours, heavy lifting, emotional labor, and histories of endometriosis and uterine myoma were associated with selected outcomes. These findings support a multidimensional approach to pregnancy-related occupational risk assessment rather than reliance on shift status alone. Pregnancy-supportive workplace policies should include timely, individualized work adjustment.

## Figures and Tables

**Figure 1.**
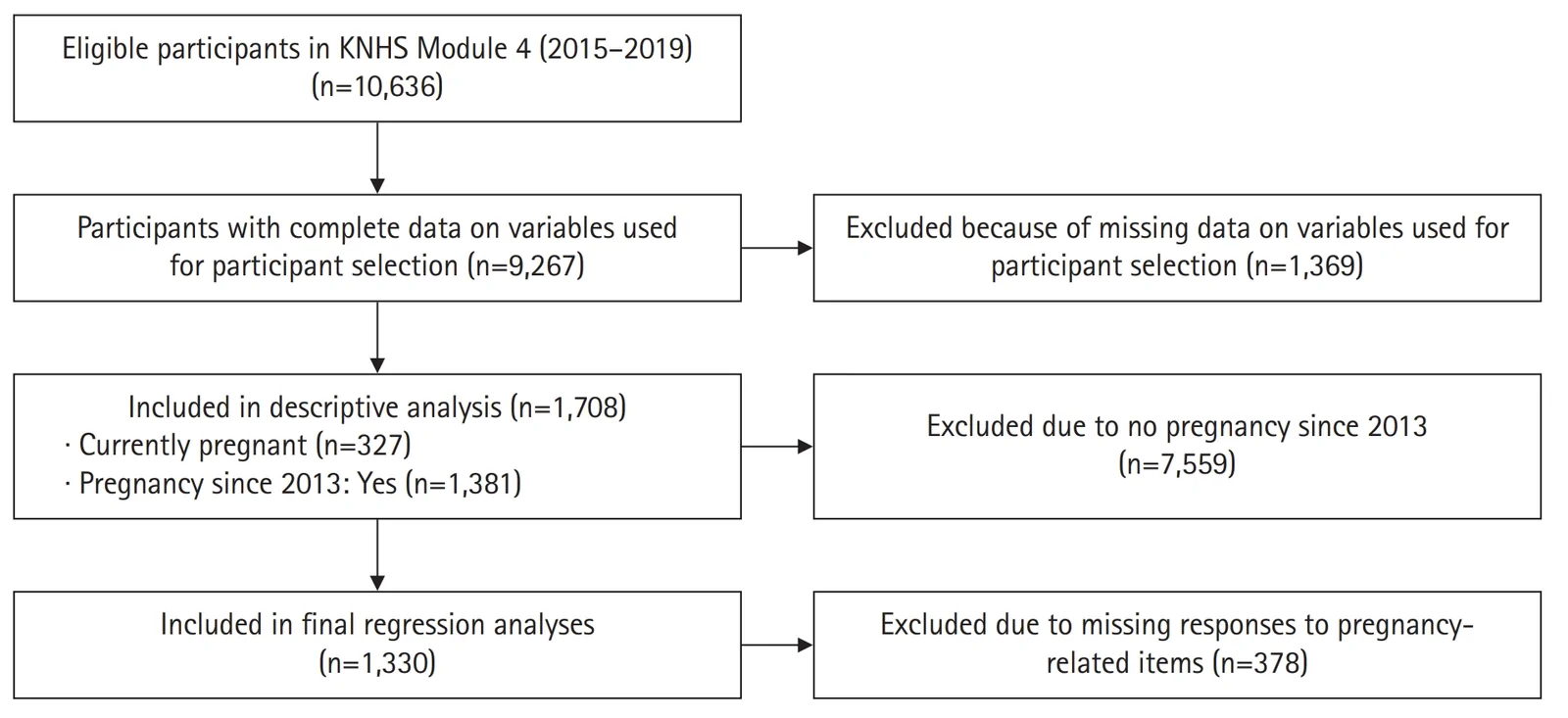
Flow chart of participant selection. KNHS: Korea Nurses' Health Study.

**Table 1.** Participant characteristics according to shift work type (N=1,708)

Variable	Mean±SD or n (%)			χ² or F	*p*-value
	Non-shift^ᵃ^ (n=883)	Shifts without nights^b^ (n=133)	Shifts with nights^c^ (n=692)		
Age (year)	33.20±3.70	32.69±3.27	31.92±3.48	24.81	<.001 (a>c)^†^
<35	728 (82.4)	104 (78.2)	654 (94.5)	59.81	<.001
≥35	155 (17.6)	29 (21.8)	38 (5.5)		
Hospital bed size					
<100	172 (19.5)	17 (12.8)	43 (6.2)	75.72	<.001
100-599	343 (38.8)	47 (35.3)	241 (34.8)		
≥600	368 (41.7)	69 (51.9)	408 (59.0)		
Overtime hours (hour/week)	2.64±3.19	3.77±3.31	4.36±3.46	53.12	<.001 (b,c>a)^†^
Overtime work					
No	274 (31.0)	20 (15.0)	51 (7.4)	137.12	<.001
Yes	609 (69.0)	113 (85.0)	641 (92.6)		
Standing time (hours/day)					
<1	273 (30.9)	19 (14.3)	31 (4.5)	207.67	<.001
1-4	297 (33.6)	34 (25.6)	245 (35.4)		
4-9	269 (30.5)	69 (51.9)	335 (48.4)		
≥9	44 (5.0)	11 (8.3)	81 (11.7)		
Heavy lifting (times/day)					
No	460 (52.1)	37 (27.8)	100 (14.5)	259.48	<.001
1-5	314 (35.6)	63 (47.4)	428 (61.8)		
6-15	62 (7.0)	26 (19.5)	124 (17.9)		
≥16	47 (5.3)	7 (5.3)	40 (5.8)		
Emotional labor score	5.92±2.40	6.72±2.04	6.67±2.03	24.68	<.001 (b,c>a)^†^
Endometriosis history					
No	867 (98.2)	132 (99.2)	675 (97.5)	1.96	.375
Yes	16 (1.8)	1 (0.8)	17 (2.5)		
Uterine myoma history					
No	813 (92.1)	125 (94.0)	649 (93.8)	1.98	.371
Yes	70 (7.9)	8 (6.0)	43 (6.2)		

† Tukey post hoc test.

**Table 2.** Frequency of high-risk pregnancy outcomes according to shift work type (N=1,330)

Outcome	n (%)			*p*-value
	Non-shift (n=736)	Shifts without nights (n=116)	Shifts with nights (n=478)	
None	336 (45.7)	51 (44.0)	195 (40.8)	
Preterm birth	51 (6.9)	5 (4.3)	30 (6.3)	.550
Preterm labor	120 (16.3)	28 (24.1)	90 (18.8)	.102
PE/E	16 (2.2)	0 (0.0)	8 (1.7)	.252
GDM	50 (6.8)	8 (6.9)	27 (5.6)	.700
IIOC	17 (2.3)	5 (4.3)	10 (2.1)	.363
Placenta previa	15 (2.0)	3 (2.6)	10 (2.1)	.930
Placental abruption	6 (0.8)	0 (0.0)	6 (1.3)	.412
Oligohydramnios/polyhydramnios	52 (7.1)	10 (8.6)	42 (8.8)	.531
PROM	73 (9.9)	6 (5.2)	60 (12.5)	.053

GDM: Gestational diabetes mellitus; IIOC: incompetent internal os of cervix; PE/E: preeclampsia/eclampsia; PROM: premature rupture of membrane.

**Table 3.** Adjusted associations of occupational and reproductive health factors with high-risk pregnancy outcomes (N=1,330)

Variable	aOR (95% CI)^†^								
	Preterm birth	Preterm labor	GDM	Oligohydramnios/polyhydramnios	PROM	PE/E^‡^	IIOC^‡^	Placenta previa^‡^	Placental abruption^‡^
Shift work (reference, non-shift)									
Shifts with nights	0.84 (0.49–1.45)	1.01 (0.71–1.43)	0.72 (0.42–1.25)	1.12 (0.68–1.82)	1.32 (0.86–2.04)	1.30 (0.53–3.22)	1.05 (0.46–2.40)	0.91 (0.40–2.07)	1.10 (0.40–3.02)
Shifts without nights	0.54 (0.21–1.43)	1.41 (0.86–2.33)	0.89 (0.40–2.00)	1.10 (0.53–2.33)	0.50 (0.21–1.20)	0.25 (0.02–3.01)	2.27 (0.85–6.06)	1.50 (0.48–4.72)	0.40 (0.04–4.20)
Age (reference, ≥35 yr)									
<35 yr	0.47 (0.31–1.23)	0.71 (0.26–2.61)	0.41 (0.21–1.15)	1.41 (0.11–3.13)	1.01 (0.84–1.55)	1.54 (0.84–1.92)	0.42 (0.33–2.61)	0.38 (0.11–1.53)	1.71 (0.75–1.93)
Number of hospital beds (reference, ≥600)									
<100	0.78 (0.51–2.23)	1.13 (0.74–1.43)	1.09 (0.81–1.44)	0.74 (0.51–1.73)	0.85 (0.22–1.46)	0.68 (0.48–1.83)	0.59 (0.11–2.01)	1.23 (0.81–2.73)	0.33 (0.25–1.61)
100–599	0.95 (0.24–1.03)	1.34 (0.51–1.73)	1.75 (1.24–2.13)	0.88 (0.64–2.01)	0.78 (0.45–2.13)	0.68 (0.41–1.95)	0.62 (0.21–3.44)	0.45 (0.11–2.10)	1.00 (0.71–2.33)
Overtime work (reference, Yes)									
No	1.10 (0.74–2.54)	0.93 (0.62–1.73)	1.06 (0.55–2.10)	1.29 (0.41–1.93)	0.69 (0.47–1.88)	1.67 (0.11–1.83)	1.00 (0.51–1.83)	0.80 (0.21–2.15)	0.38 (0.11–1.77)
Standing work hours (hour/day) (reference, ≥9)									
<1	4.94 (3.14–5.23)	0.95 (0.31–1.45)	0.88 (0.11–1.95)	1.99 (0.81–2.13)	1.07 (0.75–2.44)	0.96 (0.44–2.53)	0.71 (0.33–2.67)	1.79 (0.85–2.17)	4.63 (0.91–4.88)
1–4	1.57 (0.71–3.53)	0.85 (0.77–3.11)	0.64 (0.60–2.75)	0.75 (0.64–2.33)	1.35 (0.70–2.47)	0.58 (0.51–3.11)	0.64 (0.53–3.45)	2.97 (0.47–3.87)	0.62 (0.55–2.83)
4–9	1.26 (0.61–3.48)	0.80 (0.54–3.77)	0.51 (0.41–2.98)	0.71 (0.55–3.01)	0.74 (0.44–2.74)	0.44 (0.36–2.88)	0.82 (0.57–3.87)	1.89 (0.59–3.03)	0.91 (0.41–2.97)
Heavy lifting frequency (times/day) (reference, ≥16)									
No	0.30 (0.11–0.86)	0.60 (0.23–2.63)	0.59 (0.31–1.73)	0.54 (0.42–2.11)	0.83 (0.24–1.99)	1.27 (0.17–2.01)	0.76 (0.20–1.58)	0.25 (0.14–2.11)	0.09 (0.07–0.53)
1–5	0.63 (0.44–3.01)	0.71 (0.37–2.76)	0.78 (0.22–1.77)	0.68 (0.36–1.78)	0.84 (0.34–2.44)	0.73 (0.44–1.74)	0.34 (0.21–1.65)	0.59 (0.11–2.61)	0.61 (0.33–1.78)
6–15	0.84 (0.11–3.13)	0.96 (0.31–2.74)	1.28 (0.44–2.81)	1.11 (0.55–2.63)	1.16 (0.47–3.01)	0.46 (0.31–2.97)	1.33 (0.22–2.59)	1.06 (0.34–2.68)	0.80 (0.43–3.02)
Emotional labor score	1.04 (0.76–2.93)	1.11 (1.06–1.84)	1.11 (0.81–1.95)	0.99 (0.74–2.03)	1.06 (0.86–1.97)	1.10 (0.69–2.45)	1.00 (0.57–2.67)	0.97 (0.84–1.94)	0.92 (0.77–2.18)
Endometriosis history (reference, yes)									
No	0.45 (0.21–2.94)	0.41 (0.33–0.64)	0.42 (0.22–2.47)	0.58 (0.15–2.89)	2.79 (0.25–2.93)	0.89 (0.34–2.25)	0.31 (0.21–2.78)	0.40 (0.35–2.13)	0.53 (0.22–1.91)
Uterine myoma history (reference, yes)									
No	0.51 (0.14–0.72)	0.40 (0.12–0.93)	1.40 (0.50–1.93)	0.80 (0.61–1.99)	0.91 (0.34–2.41)	0.52 (0.25–1.83)	1.10 (0.41–1.89)	0.36 (0.21–0.87)	1.84 (0.37–2.14)

aOR: Adjusted odds ratio; CI: confidence interval; GDM: gestational diabetes mellitus; IIOC: incompetent internal os of cervix; PE/E: preeclampsia/eclampsia; PROM: premature rupture of membrane.

† Nagelkerke R², .33;

‡ Firth regression was used for outcomes with <35 events.
